# Workflow4Metabolomics: a collaborative research infrastructure for computational metabolomics

**DOI:** 10.1093/bioinformatics/btu813

**Published:** 2014-12-19

**Authors:** Franck Giacomoni, Gildas Le Corguillé, Misharl Monsoor, Marion Landi, Pierre Pericard, Mélanie Pétéra, Christophe Duperier, Marie Tremblay-Franco, Jean-François Martin, Daniel Jacob, Sophie Goulitquer, Etienne A. Thévenot, Christophe Caron

**Affiliations:** ^1^INRA, UMR 1019, PFEM, 63122 Saint Genes Champanelle, ^2^CNRS, UPMC, FR2424, ABiMS, Station Biologique, 29680 Roscoff, ^3^INRA, UMR 1331, PF MetaToul-AXIOM, Toxalim, F-31027 Toulouse, ^4^INRA, Metabolome Facility of Bordeaux Functional Genomics Center, IBVM, 33140 Villenave d’Ornon and ^5^CEA, LIST, Laboratory for Data Analysis and Smart Systems (LADIS), MetaboHUB Paris, F-91191 Gif-sur-Yvette, France

## Abstract

**Summary:** The complex, rapidly evolving field of computational metabolomics calls for collaborative infrastructures where the large volume of new algorithms for data pre-processing, statistical analysis and annotation can be readily integrated whatever the language, evaluated on reference datasets and chained to build ad hoc workflows for users. We have developed Workflow4Metabolomics (W4M), the first fully open-source and collaborative online platform for computational metabolomics. W4M is a virtual research environment built upon the Galaxy web-based platform technology. It enables ergonomic integration, exchange and running of individual modules and workflows. Alternatively, the whole W4M framework and computational tools can be downloaded as a virtual machine for local installation.

**Availability and implementation**: http://workflow4metabolomics.org homepage enables users to open a private account and access the infrastructure.

W4M is developed and maintained by the French Bioinformatics Institute (IFB) and the French Metabolomics and Fluxomics Infrastructure (MetaboHUB).

**Contact:** contact@workflow4metabolomics.org

## 1 Introduction

Metabolomics, the high throughput analysis of small molecules in biological samples, heavily depends on data pre-processing, statistical analysis and chemical and biological annotation, which are complex, transdisciplinary processes involving both computation and interpretation (Holmes *et al.*, 2008). Since analytical technologies and protocols evolve rapidly, computational metabolomics is a field of intensive methodological research, resulting in a large volume of proposed algorithms written in various languages, making their evaluation by the bioinformatics community (including reviewers) and their chaining within ad hoc workflows by experimenters difficult (Smith *et al.*, 2013).

A few user-oriented online platforms for metabolomics data pre-processing and analysis have been described recently, including MeltDB (Neuweger *et al.*, 2008), MetaboAnalyst (Xia *et al.*, 2009) and XCMS Online (Tautenhahn *et al.*, 2012). There is, however, an unmet need for an open source and open development infrastructure which would enable developers to readily integrate new modules, compare their performances on reference datasets or download and modify the existing ones for their own research.

We have thus developed a collaborative online research resource, Workflow4Metabolomics (W4M), for comprehensive metabolomics data pre-processing, statistical analysis and interpretation. W4M is a fully open-source virtual research environment (VRE; Carusi and Reimer, 2010) built upon the Galaxy environment (Goecks *et al.*, 2010) for bioinformatics developers and metabolomics users, with minimal wrapping burden of algorithms into modules, in addition to user-friendly functionalities for workflow management. Moreover, W4M includes unique computational modules for data normalization (signal drift and batch-effect correction), multivariate analysis (orthogonal partial least-squares) and annotation (via multiple databases query).

## 2 Features

### 2.1 Framework

The VRE integrates several digital resources over many layers (hardware, software, user interfaces, documentation, tools and workflows), and is based on a High Performance Computing environment (600 cores, 100 TB). The light-weight runner technology has been added to enhance interoperability and integration of components from heterogeneous environments (Linux, Windows, etc.). Multiple workflows can be run in parallel and users can rapidly analyse large datasets: for example, the full pre-processing, statistical analysis and annotation of a cohort dataset (184 raw files, 11 Go) from liquid chromatography coupled to high-resolution mass spectrometry (LC-HRMS) was performed in 5 h, using 1% of the computational resources. Modules include wrappers from existing open-source code, and innovative tools developed with standard languages (e.g. R, Perl, Python or Java). Each tool has a web page including working examples. In addition, a help desk is provided for both users and developers. An ‘app-store’ based on the Galaxy native toolshed, ToolShed4Metabolomics, fosters the local deployment of modules, facilitates the management of developer contributions, provides wrapper templates and promotes best practice guidelines. Finally, a full W4M portable virtual machine is distributed for local installation (e.g. for development prototyping).

### 2.2 Computational tools, workflows and services

W4M currently contains 19 modules covering all steps of LC-HRMS data analysis:

- Format conversion: raw data can be converted from commercial formats (e.g. Thermo Fisher.RAW) to open formats (including mzXML, Pedrioli *et al.*, 2004, and mzML, Deutsch, 2008), via a recently developed toolshed wrapper implementation of the ProteoWizard software (Kessner *et al.*, 2008).
- Pre-processing: all wrappers of the reference XCMS (Smith *et al.*, 2006) and CAMERA (Kuhl *et al.*, 2012) functions are available to perform peak extraction, retention time alignment and annotation of isotopes and adducts.
- Normalization: signal drift and batch-effects, which are two major source of bias in MS data (van der Kloet *et al.*, 2009), can be corrected by fitting linear or local polynomial regression models to quality control samples.
- Statistical analysis: in addition to parametric and non-parametric univariate tests, W4M offers unique functionalities for multivariate modelling, including orthogonal partial least-squares (Trygg and Wold, 2002), with all numerical and graphical results and diagnostics (optimal number of components estimated by cross-validation, variable importance in projection, model significance by permutation testing, outlier detection).
- Annotation: a formula generator based on the HiRes (High Resolution) algorithm (Kind and Fiehn, 2007) is provided, in addition to several modules for public database query which allow the user to define specific annotation strategies (e.g. by searching from general to more specialized resources).

Metabolomics scientists can access W4M with a simple web browser, upload their data, select analysis parameters or choose the default settings, and run their workflows in batch mode. In addition, W4M provides functionalities for creating interactive web-based documents showing the results of the analyses, and sharing them with collaborators directly on W4M. To get started easily, pre-configured workflows and corresponding histories are publicly shared for pre-processing, statistical analysis and annotation, respectively. A real LC-HRMS dataset (Roux *et al.*, 2012) is provided as a reference for new module and workflow evaluation.

## 3 Conclusion

The W4M infrastructure enables both experimental users with no specific programming skills and advanced developers to perform cutting-edge and reproducible computational analyses from raw data to metabolite annotation. W4M can be further extended to integrate external workflows running on desktop platforms (e.g. Taverna, KNIME), or acquisition instruments. The statistical modules from W4M can be used to analyse other ‘omics’ data, or can be combined with existing Galaxy workflows (e.g. in transcriptomics), thus enabling multi-omics analyses in a global systems-biology approach. In the coming months, modules for NMR data pre-processing will be integrated into W4M and the infrastructure will be connected to MetExplore (Cottret *et al.*, 2010) for genome-scale network analysis. W4M is therefore an innovative open-source computational VRE bridging the data-intensive bioinformatics and metabolomics communities.

## Funding

This work was supported by Biogenouest®, Lifegrid (Auvergne), and by the IDEALG project [ANR-10-BTBR-04], IFB [ANR-11-INBS-0013] and MetaboHUB [ANR-11-INBS-0010] grants.

*Conflict of Interest*: none declared.
